# Towards a mother-centred maternal health promotion

**DOI:** 10.1093/heapro/daad014

**Published:** 2023-02-25

**Authors:** Eva Neely, Anna Reed

**Affiliations:** School of Health, Te Herenga Waka—Victoria University of Wellington, PO Box 600, Wellington 6140, New Zealand; School of Health, Te Herenga Waka—Victoria University of Wellington, PO Box 600, Wellington 6140, New Zealand

**Keywords:** maternal health promotion, Ottawa Charter, motherhood, equity

## Abstract

A transformative approach to maternal health promotion should be mother-centred, context-driven and grounded in lived experiences. Health promotion can achieve this by drawing on its disciplinary roots to extend and reorient maternal health promotion towards an approach of non-stigmatizing and equitable health promotion that has mothers’ well-being at the centre, particularly giving credit to marginalized, ‘non-normative’ maternities. This article draws on data from 18 workshops EN conducted across Aotearoa New Zealand, including 268 maternal health stakeholders. Drawing on design thinking, participants reimagined what a maternal health promotion approach informed by the Ottawa Charter action areas could comprise. The five themes included building connected systems close to home, developing mothering/parenting skills, addressing upstream determinants, mother-centred care and funding, and creating a collective mothering village. We discuss how these areas could better meet the unique challenges of transitioning to motherhood. Rather than focussing only on individual behaviours, many ideas reveal broader environmental and structural determinants. We link the themes to current literature and advance the agenda for centring the maternal in maternal health promotion.

## INTRODUCTION

Globally, maternal (we note here that not all mothers and birthers are cis-gender women. We use maternal and mother to represent the health needs of those who give birth to babies whilst acknowledging the unique needs of women and gender diverse birthing populations that cannot be conflated. We see it as pivotal to include gender diverse needs in reproductive health into a maternal health strategy that can sit alongside and collectively advance justice agendas) health needs are currently not being met appropriately, and require improved resourcing and advocacy (Koblinsky *et al.*, 2016). Socio-political, environmental and demographic changes are likely to contribute to transforming needs in maternal health into the future. This transformation, alongside urbanization, information overload and rising expectations for care, requires a comprehensive, multifaceted approach (Kruk *et al.*, 2016). Perinatal mental health issues (Howard and Khalifeh, 2020), growing inequities (Crear-Perry *et al.*, 2021), barriers to care (Dawson *et al.*, 2019), as well as a rise in birth interventions (Fox *et al.*, 2019; Betran *et al.*, 2021) are impacting maternal health. Additionally, perinatal care is insufficient for meeting the needs of trans and non-binary people (Malmquist *et al.*, 2019). However, optimal maternal health is critical for a resilient and thriving society, with experts arguing that a more comprehensive population health approach to maternal health is needed (Kruk *et al.*, 2016; Miller *et al.*, 2016). A health promotion approach has the potential to advance such an agenda with its focus on equity, social determinants, community participation and environments for health (Mittelmark *et al.*, 2008).

Maternal health promotion has been shaped by neoliberal values and deep-seated patriarchal understandings of women’s bodies, contributing to increased inequities, surveillance and victim-blaming of mothers (Ayo, 2012; Lupton, 2012). Maternal bodies have increasingly become the site of control for ensuring expectant mothers closely monitor their health behaviours, placing their unborn’s well-being above their own. Such maternal sacrifice is crucial for being a ‘good’ mother in the 21st century and requires mothers to entirely focus on their baby’s needs (Elliott *et al.*, 2015). This emphasis effectively neglects and diminishes the *maternal* transition, despite being one of the most impactful life changes (Wadephul *et al.*, 2020).

In this paper, we will expose the tacit assumptions often embedded in maternal health promotion and discuss what a values-driven health promotion approach offers. We will enrich this with empirical data from maternity stakeholder workshops for imagining the opportunities of a comprehensive and multi-level maternal health promotion approach.

### The neoliberal neglect

Gender inequity is a pressing global challenge to which health promotion has not responded sufficiently (Sen and Östlin, 2009; Pederson *et al.*, 2014). Although gender is an acknowledged determinant of health (WHO, 1986), it is not sufficiently recognized in prominent health promotion frameworks and strategies (Gelb *et al.*, 2012; Fisher and Makleff, 2022). This gender-blind approach significantly impacts mothers who often face intersectional dimensions of inequities (Vandenbeld Giles, 2014; O’Reilly, 2016). In order to reduce the inequities mothers experience, we need to change the approach to comprehensively promote health across the perinatal period (Wadephul *et al.*, 2020).

Health promotion has long been under the auspices of neoliberalism, championing and reproducing the self-regulating healthy body as a moral obligation (Ayo, 2012); translating into a perspective of maternal health where society views women in their reproductive years as incubators for future healthy citizens (Raphael-Leff, 1991; Lupton, 2012). Positioning childbearing women as morally responsible for producing an economically viable future generation creates immense pressure for uncontrollable outcomes, yet is sold as enabling choice and self-determination. Thus, while neoliberalism ‘appears to “emancipate” all people within a discourse of “equality”, in reality, it further entrenches inequalities by obscuring structural factors of poverty, gender and race’ (Vandenbeld Giles, 2014, p. 417). This emphasis on freedom of choice and self-fulfillment marginalizes mothers without the resources to ‘make it on their own’ (Ware *et al.*, 2018; Parton *et al.*, 2019; Neely *et al.*, 2023). Accordingly, the dominant approach to promoting mothers’ well-being emphasizes goal-oriented, individualistic and instrumental values.

### Maternal health: a means to an end

The neoliberal agenda has envisaged pregnancy as a window of opportunity to intervene and minimize health risks (Ayo, 2012; Peterson and Lupton, 2012). The linkage of maternal lifestyle behaviours and body shapes, such as ‘obesogenic’ wombs (Parker, 2014), has led to a proliferation of research seeking to shape health trajectories from the womb (albeit mostly limited to lifestyle behaviours). The inordinate focus on health behaviours during pregnancy has prevailed for decades resulting in public health messages that moralize and define ‘good mothers’ (Lupton, 2012). Such messages are not prioritized as a matter of the cause (women’s health) but to monitor behaviours to produce healthy offspring. Women are targeted for practices, including eating safely and healthily, not smoking or drinking and managing stress, and this is not even bound to the realm of ‘actual’ pregnancy but extends to any woman of childbearing age as ‘pre-conception’ care (Lupton, 2012; Hallgrimsdottir and Benner, 2014; Waggoner, 2015). The failure to attend to mothers’ needs perpetuates gender inequity by falsely giving the impression that maternal health is on the agenda. This trend can be placed within a broader global perspective in which women’s health is often not prioritized and poorly resourced (Spong, 2020; Vechery, 2021).

Further evidencing the missing authentic motivation to promote women’s health is the lack of action on non-communicable diseases, despite these now posing one of the most significant burdens of death worldwide for women (Vogel *et al.*, 2021). Thus, the underlying current for maternal health promotion we observe can be explained by the fact that women’s health is neglected unless their bodies are reproducing, in which case monitoring, surveillance and control are warranted to maximize the future health of our citizens. Accordingly, calling for a mother-centred health promotion includes a call to action to prioritize women’s health for its own sake, as of value in and of itself.

### Mother-centred maternal health promotion

Health promotion strives for upstream approaches that are socially proactive and committed to positive health for all. A values-driven approach to health promotion embodies the core values of holistic, salutogenic and ecological health, alongside social justice and equity (Gregg and O’Hara, 2007). We discuss some fundamental tenets of these values in relation to motherhood here but refer the reader to Gregg and O’Hara (Gregg and O’Hara, 2007) for a more elaborate discussion on health promotion values. Viewing motherhood from a health promotion perspective enables us to consider *holistic* health needs that push the boundaries of pre-defined parameters of health change. We can more openly address and consider the affective, emotional, physical and spiritual aspects of motherhood, alongside viewing the reciprocity and impact of social relations as pivotal to health (Wadephul *et al.*, 2020). An *ecological* lens considers the complex interaction of individual, community and societal level factors that together produce health to meet the contextual demands of diverse realities. Such a perspective then also deems the ability to mother as dependent on socio-political determinants of health, in which inequalities exacerbate women’s adverse outcomes and demand addressing. A *salutogenic* perspective offers the opportunity to focus on health and flourishing over illness and disease. Given that pregnancy, birth and motherhood are not a disease, but a normal life stage, a salutogenic (strengths-based) perspective is spot-on in retiring pathologic and deficit framings (Perez-Botella *et al.*, 2015). Core to promoting maternal health is a commitment to *equity* and *intersectional reproductive justice* (Ross and Solinger, 2017) to depart from centring white-cis-hetero mothers in maternal health promotion. Additionally, as a colonized country, Aotearoa is committed to transforming health inequities between indigenous (Māori) and non-indigenous people through bicultural praxis (Came and Tudor, 2016).

Positively, values-based health promotion offers a more distributed agency, which means we can think of the maternal transition as comprised of shared responsibility across a range of agencies (Neely, 2023). Becoming a mother then overtly happens in reciprocity with the environment: this demands from us, as a society, that we optimize the conditions across individual, community and policy levels that enable mothering. We consider health needs holistically, foster maternal participation and voice in determining the care and support we give, and underscore every action we take with an intersectional equity lens that acknowledges how mothering is practised across race, gender and culture.

We also adopt a gender-transformative approach to health promotion that ‘addresses the causes of gender-based health inequalities and works to transform harmful gender roles, norms and relations’ (Pederson *et al.*, 2014, p. 143). Such an approach tackles gender norms, addresses social and structural determinants of health, including underlying gender-related determinants and draw on complex thinking (Fisher and Makleff, 2022). We extend this approach to health promotion with an intersectional and gender-inclusive perspective. We therefore situate this work within an intersectional feminist framework on mothering that considers the diverse realities of people who engage in mothering practices (Zufferey and Buchanan, 2020). Accordingly, we recognize that *being*, *doing* or *feeling* ‘maternal’ and ‘mother’ are not exquisitely available to cis-gender women. However, gendered differences attributed to parenting continue to shape caregiving practices and rather than use gender-neutral terms (parental/parent) we use gendered terms (‘maternal’ and ‘mother’) to signal this where appropriate. Taking up the challenge of not conflating social categories with disadvantage, but preserving an intersectional equity lens, we locate the core task of our work as residing in mothering and maternal practice, rather than binding such practice to any particular group. We seek justice for those who birth babies, care for babies, and do the grunt work as caregivers of small children. In doing so, we wish to acknowledge that the majority of care labour lies on the backs of women, which needs addressing, while simultaneously seeking to pursue maternal health promotion for others who birth and/or provide childcare (e.g. fathers, family members, sexual minority parents and parents of diverse genders).

We also draw on matricentric feminism (O’Reilly, 2016), which acknowledges that feminist advancements over the past decades have left mothers behind, and considers the challenges and contexts of mother-centred feminist theory. Matricentric theory adopts mothers’ concerns and needs as a point of departure for developing a theory and politics of conditions that empower those who mother.

### Ottawa Charter

The Ottawa Charter is a defining document that laid the groundwork for modern health promotion (WHO, 1986). The Charter embodies the core values we just discussed and provides a framework for considering action across multiple levels. The Charter has eight prerequisites for health (peace, shelter, education, food, income, stable eco-system, sustainable resources, social justice and equity); three strategies (enable, mediate, advocate); and five action areas for improving health (build healthy public policy, create supportive environments, strengthen community action, reorient health services and develop personal skills). Since its inception, there have been critiques and benchmarking exercises to determine its usefulness for health promotion in the 21st century (McPhail-Bell *et al.*, 2013; Thompson *et al.*, 2018; Wilberg *et al.*, 2021). We acknowledge its shortcomings; however, we value its ease as a practical tool for research engagement to think about multi-level health promotion. For this research, we drew on the five action areas to offer a model for imagining comprehensive mother-centred maternal health promotion.

### Maternity system in Aotearoa New Zealand

Maternity care in Aotearoa is publicly funded through a midwife-led continuity model of care. The system is built on a partnership model of care in which care is enacted through trust, respect and shared decision-making (Guilliland and Pairman, 1994). Midwives provide community-based primary maternity care to 95% of the population (Te Whatu Ora, 2022). Women/birthing people can choose their location of birth (hospital/birthing centre/home) though there is regional variation in access to appropriate care and facilities. Obstetric care is available through private health care expect for high-risk pregnancies for whom such care is funded.

## METHODOLOGY

This research adopted a participatory inquiry approach (Burns, 2012), drawing on aspects of design thinking (Brown and Wyatt, 2010) to collect diverse voices on what a complex solution to a maternal health promotion strategy might comprise. Participatory inquiry involves multiple stakeholders to gain insights into the ‘convergence and divergence’ about what is going on (Burns, 2012). This explanation is congruent with the aims of this study, which is to look at different stakeholders in the field of maternal health to understand needs as framed through a health promotion lens. Participatory approaches, also known as co-design, are more solution-focussed than other forms of research and are appropriate in using contextual data and expert opinions to formulate a strategy for a complex health problem. Design thinking aids with this by fostering the ability to ‘dream’ and transcend the ‘immediate boundaries of the problem to ensure that the right questions are being addressed’ (Panke and Harth, 2019, p. 195) and surpass constraints. In this process, the facilitator engages with participants in creation, synthesis and divergence to imagine what is possible.

Burns (Burns, 2012) states that research needs to ensure that ‘the diversity of the whole system is represented (as much as is possible), that there are multiple potential entry points for action, and that there is engagement from people with different interests across the system’ (p. 90). Through the engagement with diverse stakeholders and the Ottawa Charter action areas as a model to facilitate thinking across multiple levels of action, we sought to explore the potentialities of a mother-centred maternal health promotion framework. This research was approved by the Massey University Ethics committee.

### Workshops

We hosted 18 workshops around Aotearoa. EN gathered the data using the Ottawa Charter action areas, which she explained to participants using the World Health Organization descriptions (WHO, 1986). Participants started by mapping what kind of maternal health services and resources they had in their community, which acted as a stocktake activity to help them see the strengths and gaps in their community (data not included in this study). Participants were then asked to envisage maternal health promotion in an ideal world and invited to dream beyond boundaries. Participants were given post-it notes and moved around the room between five big A1 sheets of paper, each named with an Ottawa Charter action area. Consequently, participants walked around with stickers to prioritize actions they considered most pivotal. Participants conversed in these spaces, and there was a ‘buzz’ as they brainstormed what they wished for in maternal health promotion. No identifying information was given about who said what. A research assistant typed the data from the workshops up into excel sheets.

### Participants

‘Stakeholders’ in this research included many health professionals, charitable organizations and mothers. There were 268 participants, including consumers (mothers), midwives, lactation consultants, alternative health providers, birth education providers, doctors/obstetricians and a few politicians. Of the 18 workshops, 8 were in larger urban centres, and 10 were in smaller towns situated more rurally. All participants had either a personal or professional (or both) interest in maternal health, with the majority involved in supporting mothers/parents currently.

### Analysis

We used thematic analysis (Braun and Clarke, 2006, 2021) to analyse the data. AR started by descriptively coding responses in each action area using NVivo. Subsequently, we examined codes for similarities and divergences and worked those that matched into sub-themes. For example, ‘more consumer voice’, ‘consumer advocacy’, ‘consumer network’ and ‘co-design’ conglomerated around ‘strengthening consumer voice’. We then went back and forth between authors to determine the fit and distinguish themes resulting in the five themes we produced from the data. Participants are not named, and it is not possible to correlate views with people.

## FINDINGS

We generated five themes from the vast amount of data and ideas developed in the workshops. The Ottawa Charter loosely guided our analysis, but we drew on inductive theming to break outside those boundaries. The five themes included building connected systems close to home, developing mothering/parenting skills, addressing upstream determinants, mother-centred care and funding, and creating a collective mothering village. Words taken from the participant mind maps are italicized.

## BUILDING CONNECTED SYSTEMS CLOSE TO HOME

Participants across most workshops desired a more *connected, local* and *culturally responsive* system. It was telling that people from the same community met for the first time at the workshops and learnt about related services they never knew existed. Participants saw maternal services as *disjointed*, *medicalized* and *inaccessible*.

Across workshops, it was evident that communities wanted to set their own goals, aiming for maternal health to be *community-driven* and *community-owned*, recognizing that centralized decision-making was not in the best interest of communities. Community-owned services based on need were seen to serve the diverse range of communities in Aotearoa better, and it was recognized that *what works for one community will not work for another*. Many participants mentioned *trust* as a reason for wanting community-driven solutions and setting their own health goals. Particularly for Māori communities, participants indicated that centralized power exerted through external government agencies and colonial legacies has led to distrust. Participants also specified that maternal health systems should be *culturally responsive*, such as locating more services within *marae* [Māori meeting grounds] *and Pacific churches*, using *Māori health models* (e.g. *Te Whare Tapa Whā)* and adopting a *holistic view of health*. Participants sought health services to be *strengths-based and empowering* and link identity and culture (*Te Ao Māori)* to health services. Such sentiments resonate strongly with the desire and urgent need to orient maternal health services and systems to bicultural models with Indigenous leadership (Came and Tudor, 2016; Stevenson *et al.*, 2016; Ware *et al.*, 2018).

Participants in the workshops discussed the desire for local systems that are *collaborative* and *connected*. Many participants felt that current service provision is *disconnected, fragmented* and *reliant on individuals* rather than anchored in co*llaborative relationships and services*, moving away *from silos to information sharing*. Participants noted that collaboration would provide *consistency* and *coordination* between services, which would be positive for maternal outcomes. New Zealand has a history of ‘fragmented’ systems (Cumming *et al.*, 2021) in which the ‘barbed-wire fence’ ringfences medical and ‘non-medical’ primary care and stands in the way of meaningful social and health sector integration (Tenbensel *et al.*, 2017). A review on maternal mental health found that ‘innovative and dynamic’ initiatives adapted to the local context and involving self-determination, participation and partnership are more likely to succeed (Dawson *et al.*, 2019). There is a move toward locally adapted system approaches in health promotion to address complex health issues (Matheson *et al.*, 2018), which could be effectively drawn upon to address some critical challenges encountered in maternal health.

A common opinion expressed that mothers are best cared for *at home and in the community*, *not in DHB* [district health board] *buildings.* Participants also noted the physical and financial barriers to accessing care if services were not close to home. Physical access to care for rural and urban mothers/parents in Aotearoa, including travelling for appointments, finding childcare and transport, is a problem (Dawson *et al.*, 2019; Neely *et al.*, 2020). Some participants said that *all child and maternal service providers should be located in a hub*, making it easy to access services and information about providers. Some people talked about *co-location of services*, but for many, the hub concept went beyond this. Participants also suggested that a hub could offer free counselling and *wāhine ora* (women’s well-being) checks to reduce financial and physical access barriers. Participants were particularly concerned about *accessible, less bureaucra*tic and *safe* services. Participants suggested a *one-stop local shop for everything parenting*-*related*, including alternative health providers, such as naturopaths, osteopaths and lactation consultants. An example included *The Loft*, a co-location of health and social services, which aims for accessibility by offering walk-in services that can be wrapped around a person quickly (The Loft, 2022). Priday and McAra-Couper (Priday and McAra-Couper, 2016) discuss the benefits of midwifery co-location with other healthcare providers in high deprivation, highlighting how it enables multidisciplinary teams and reduces barriers to care access.

Along with community-based care close to home, a theme explored in the workshops was the idea of safe spaces that are not tainted with negative healthcare experiences but where mothers find *support* and *compassion*. One quote highlighted the importance of *programmes in safe facilities in spaces not overshadowed by probation or Oranga Tamariki* [child welfare agency] *visits.* Community-based services are more likely to be trusted than medical facilities (Stevenson *et al.*, 2016), adding to the justification for more community-led maternity services and spaces.

## DEVELOPING MOTHERING/PARENTING SKILLS

Participants highlighted the significant transition and transformational time of becoming a mother and the skills required. Participants’ ideas around developing mothering/parenting skills included *rethinking antenatal education*, *providing more breastfeeding education and support*, *understanding the wide range of ‘normal*’ in parenting, *accessing credible parenting information*, *empowering professional relationships, skills in schools and parenting education*.

Participants noted a need to reconsider antenatal education towards greater *inclusiveness* and *relevance*. For instance, a greater emphasis on *birth rights* and *informed consent* were desired. *Financial* and *physical access barriers* and discrepancies were also discussed as determining inequities in antenatal education. A broader offering of antenatal classes was seen as helpful for offering diverse and accessible options. Renkert and Nutbeam (Renkert and Nutbeam, 2001) recognized over 20 years ago that antenatal education should shift towards antenatal literacy, however participants thought there was still a surprising scarcity of classes that meaningfully address information needs of the 21st century. Additionally, there is still much room to make antenatal education more gender-inclusive (Ritchie and Lai-Boyd, 2022) and culturally specific (Ware *et al.*, 2018; Shrestha-Ranjit *et al.*, 2020). Given that human connection and friendships are a core part of antenatal education (Spiby *et al.*, 2022), it is also essential to consider the effects a shift to online classes in the digital era could have.

Breastfeeding support was a prominent concern across the data, such as *support* and *demonstrations antenatally*, *drop-in centres* and *more funding for lactation consultants* to encourage sustained breastfeeding. Many participants wanted *milk banks* to be available. Participants also discussed the importance of positive, supportive environments for breastfeeding. They suggested *breastfeeding friendly cafés* as examples of positive environments, *breastfeeding positivity* in the media to make it more visible and accepted, and p*ositive role models or celebrities* to model and normalize breastfeeding. A public health approach to breastfeeding that recognizes environmental factors and addresses broader education and better postnatal support (Brown, 2017) remains a missing cornerstone of maternal health promotion.

Access to, and literacy of, parenting information was seen as lacking. Parents have access to a broad scope of information, especially online. Participants thought it was difficult for many to decide which information was trustworthy and where to start reading, suggesting a *one-stop-shop to find parenting information*. Participants proposed a nationwide service for accessing information, perhaps online, and *both consumers and healthcare providers know where to find this information*. *Finding antenatal providers*, mother’s groups, support groups or maternal health providers is *not straightforward and can be difficult*. Some participants expressed concern about an *overload of information* leading to anxiety and the difficulty of retrieving appropriate information on parenting that is *inclusive* and *easy to identify*. Participants saw the need for *easy-to-access evidence-based parenting resources* and called for a move to *evidence-based government-sponsored apps*, with a *low literacy level* needed, to ensure that information is accessible and evidence-based. Lupton (Lupton, 2016) found that there is ‘little to no regulation’ (p. 2) on what information is available on apps as well as on social media. This means that the quality of information is variable and relies on individual resources and literacy. Recent developments in Aotearoa has led to some Indigenous-lead breastfeeding and parenting apps that fill some of the gaps discussed (Hāpai Te Hauora, 2023; Wray, 2023).

A core theme in the workshops was the narrow view of ‘normal’ in pregnancy, birth and parenting. Participants discussed the need for mothers/parents to understand *the normal variation in baby sleep, diet and baby habits to more easily recognize ordinary parenthood experiences instead of attributing their feelings to mental health*. Given the transformative nature of parenthood, it could be easy for a new mother/parent to assume that their feelings or baby’s behaviour are abnormal. Recognizing that ‘normal’ has a considerable variation could help with anxiety and isolation. In Aotearoa, child health checks were said to be more focussed on developmental timelines rather than the needs of individual families, which can propagate this fear. Supporting skills and information sharing of a broader scope of ‘normal’ was a critical component of maternal health promotion. Investing time and research into developing practical communication skills (Cutajar *et al.*, 2020) to support a greater ‘scope of normal’ could be a step forward.

Across the workshops, a unanimous response from participants was to focus on schools as environments where young people can learn parenting, health and life literacy skills. Many participants discussed *promoting women’s health in schools early* and creating a society where people *understand more about their health and bodies*. Participants mentioned that students should learn about *Hauora* (holistic health) and that the *Ministry of Education could collaborate with health and community services on parenting and life skills*. Examples of school-based skills included *healthy relationships, normal physiology, health literacy, sex education, budgeting and life skills*, which all affect the role of the parent. Schools are valuable health-promoting settings within health promotion, and extending areas relevant to pregnancy, birth, motherhood and parenting warrant greater attention.

## ADDRESSING UPSTREAM DETERMINANTS

Participants identified several upstream (social, structural and economic factors that impact health) changes that should be incorporated into a maternal health promotion strategy, including *healthy homes*, *paid parental leave, affordable living and family–friendly workplaces*.

Participants raised the importance of *warm, safe, healthy, affordable* and non-*overcrowded housing* for maternal health, and put forward a ‘*warrant of fitness’* for housing. As a new parent, the home is the primary occupation space—many venture little outside their walls in the first weeks/months of parenthood. Unsafe, insecure and damp housing has well-established links to adverse socio-emotional and physical health outcomes (Rolfe *et al.*, 2020). For mothers/parents at such a vulnerable time, insecure and inadequate housing has multifaceted, complex and intergenerational effects on health (Reece, 2021) and destabilizes their ability to adjust to new challenges of mother/parenthood. The idea that housing is linked to adverse health is not new, albeit unresolved and under-addressed; however, linking housing as a core strategy within a coordinated maternal health promotion approach crucially steps away from remaining at more widespread behavioural approaches to improving maternal health. The explicit recognition of this deficiency in maternity by stakeholders across the maternity field indicates the observed effect such housing has on maternal health.

Participants expressed a need for extended, paid leave for both parents to enable bonding and support for the whole family. Participants specified *12 months’ paid leave minimum, paid support partner leave* and leave for *grandparents* or a *nominated support person*, recognizing the diversity of family structures (in Aotearoa, paid parental leave entitlement is currently at 26 weeks). Longer paid leave can improve parental health and facilitate attachment and breastfeeding duration (Cooklin *et al.*, 2012; Bilgrami *et al.*, 2020). Reilly and Morrissey (Reilly and Morrissey, 2016) also recognize that not all families are nuclear and that the partner of a primary caregiver could be adopted for leave entitlements to ensure equity and flexibility. Having a present co-parent at home is positive for maternal health and can reduce the isolation often experienced in motherhood (Lupton, 2016). Partner parental leave would also work towards gender equity and allow partners to be more actively involved in the early care of their children, helping with bonding and attachment and playing an essential part in promoting maternal health (Reilly and Morrissey, 2016).

Participants were concerned about affordable living in the perinatal period. They observed how families struggled to afford basics, including *food, transport* and *power bills*. Increased time at home and use of household items meant increased bills. Preexisting inequities were thought to be exacerbated upon the arrival of a baby. Gender inequities further compound such disadvantage where women are more likely to be in low-paid, insecure work and have less access to money and transport (McLeish and Redshaw, 2019a; Neely *et al.*, 2020). Participants considered actions such as *food vouchers, living wages, universal income,* and *tax-free fruit and vegetables.* Integrating upstream initiatives into a maternal health promotion strategy holds promise, with interventions such as food subsidy programmes (McFadden *et al.*, 2014) being a first step towards acknowledging this shortcoming, yet not enough to address the systemic and structural changes needed (Dawson *et al.*, 2022).

Flexible and family–friendly workplaces were also seen as a crucial step towards equity by enabling better integration of paid and unpaid work. This included a plea for *adequate and pleasant breastfeeding or pumping spaces and breastmilk storage*, *integration of work and family*, *flexible hours*, *job sharing* and *part-time work* options. Common factors affecting maternal health and well-being concern balancing paid and unpaid employment, where workplace structures inhibit cumulative and cause daily, accumulative stress to everyday life (Hokke *et al.*, 2021). Advocating for greater flexibility in accommodating family life needs, which disproportionately affects women and LGTBQI+ parents (King *et al.*, 2013), could play a central role in reducing daily stress, particularly focussing on formal over informal policies (Hokke *et al.*, 2021).

## MOTHER-CENTRED CARE AND FUNDING

Participants were clear that the current system was not mother-centred and perpetuated inequities and considered perinatal physical and mental health needs requiring high resource investments, including *time, money* and *initiative*. Participants advocated for equitable and accessible services such as:

*delivering better mother-centred care postpartum, individualised funding, free GP visits, funding birth trauma support and counselling, flexible care and more focus on mental health.*

Many participants talked about the apparent lack of support for mothers post-birth. With a very infant-focussed system, mothers’ physical, emotional and social needs are often neglected and only supported in resource-rich settings, *which reinforce existing inequities*. *Doulas*, *home help*, *equitable early discharge* and *support at home* to help with *meals, laundry* and *nappies* were mentioned as effective support mechanisms to ease the transition to motherhood. However, access to such support *was inequitable given the cost*. Participants also considered the absence of mothers in initiatives that seek to improve child health (e.g. ‘the first 1000 days’) unacceptable (for instance seen in nutrition interventions, Kinshella *et al.*, 2021). Bringing a mother-centred lens to such issues could feed a ‘maternal health in all policies’ approach. O’Fahey and Shanessa (O’Fahey and Shanessa, 2013) also highlight that mothers are neglected in the postpartum period and develop a perinatal maternal health promotion model that seeks to help mothers develop four critical individual health-promoting skills: the ability to mobilize social support, self-efficacy, positive coping strategies and realistic expectations. Our data shows that such supports are essential for maternal well-being; however, the structural support required to foster such an approach is lacking. Further, individual skills can only be one component of effective health promotion.

Participants considered it important to reorient and *fund a person rather than a service,* to recognize different needs. Access to funding for postpartum needs could address some of the gendered health inequities that result from giving birth. Participants proposed straightforward solutions to this, such as *extending the current Best Start package* to include a *postpartum fund* that mothers/parents could use for their individual needs and circumstances. Participants suggested *funded pelvic floor physio checks*, *alternative and complementary health providers, traditional Māori healing, postpartum doulas* or items including *food, medicine and petrol* as examples of services that could benefit mothers/parents postnatally. Such funding would allow more tailored access to resources and reduce the burden of and barriers to seeing a general practitioner. Person-centred funding would also allow for greater access to culturally tailored services. Further underscoring this point, participants emphasized how funding the person would allow the embracing and valuing of *multiple knowledges* that could help work towards decolonizing maternity care.

Alongside person-centred funding, there was a desire to have *free GP visits*, *counselling, physiotherapy, reprioritisation of funding for women’s health, regular health visits* and *funded birth trauma therapy* to ensure that the postpartum mother is not forgotten. Overwhelmingly, participants recognized the extra cost in the perinatal period and the resulting gender inequities. Participants also mentioned *funding visits up to 1–2 years postpartum* to ensure that the mother has accessible avenues to discuss any physical or mental health concerns that might come up after the initial postnatal period is over. Health care costs, compounded by transport, time scheduling and prioritization of child needs, present key barriers to access for postpartum needs (Lee and North, 2013). Recently (October 2022) introduced cover of maternal birth injuries in Aotearoa is one step towards improved funding for physical impacts from birth (ACC, 2022), however in this paper we hope to invite a broader upstream and salutogenic approach to thinking beyond biomedical needs in maternal health.

Participants emphasized the severe need for *maternal mental health support* because the current system was *under-resourced, hard to access* and *focussed on high needs, not early intervention*. Maternal mental health services and supports are in greater demand across countries yet collectively indicative of the gaps in structural approaches to maternal health promotion (Shuffrey *et al.*, 2022). Whilst maternal mental health services are crucial for mothers/parents with clinical mental illness, drawing more on group-based community support systems or peer support programmes could meet a high amount of need and be efficient, cost-effective and sufficient tools for improving maternal mental health (Atif *et al.*, 2015).

Participants highlighted the need for more consumer voice as central to mother-centred care, particularly for hard-to-reach mothers/parents to inform co-design of services. The sheer amount of responses in this space showed that mothers did not feel heard. Participants desired a greater emphasis on *reaching consumers*, *getting consumers on board* and *consulting consumers for priorities.* Consumer voice surveys and maternity care feedback are useful; however, they did not meet the desire for *canvassing collective voices* or *accountability for action* in response to consumer voice. This observation resonates with issues of tokenistic representation in mental health consumers (Scholz *et al.*, 2019). Participants suggested that consumers should also be involved in *co-designing services and asked for their priorities* and *meaningful engagement*. Other participants focussed less on individuals but more on *finding out what a community wants* and its *priorities.* There was also a desire for a *strong consumer network* where *every consumer has a point of contact* and mechanisms to ensure these voices are heard. Consumer representative groups were seen as good but insufficient to engage in meaningful consumer voice. Dawson *et al.* (2019) assert that while maternity experience surveys in Aotearoa show us how mothers experience the current system, this does not tell us what could be improved. Meaningful consumer engagement takes time, resources and enduring relationships (Kennedy, 2008) and requires more than self-selected representatives for a large group of diverse birthers. Authentic and meaningful work with consumers is vital for this role to empower and effectively inform inclusive and equitable service design (Scholz *et al.*, 2019).

## CREATING A COLLECTIVE MOTHERING VILLAGE

The need for a mothering village with community and social support was a central theme across workshops. Participants had numerous ideas of what such a village would look like and overwhelmingly favoured *face-to-face support* over online (notably, this data were collected before the COVID-19 pandemic). Participants noted various mechanisms that acted as a village, such as *coffee groups, mother–baby-playgroups* or *baby-friendly cafés*, which, however, often were seen to rely on *mobility, financial privilege* and *confidence* in public spaces. Broader yet related suggestions included links to the local context, such as *getting to know neighbours* and *street parties*, indicating how separated people feel from their own neighbourhood.

Many contributions centred on improving physical environments by removing barriers to access. Ideas included having

*physical hubs, family centres, pram-friendly footpaths*, *spaces made for families, appropriate urban design for mums and bubs* and *community gardens* (related to hubs in the first theme but extended to more functions than health services).

Such a lack of available places for mothers can be seen as a ‘spatial expression of patriarchy’ (Valentine, 1989, p. 389) in which powered gender relations confine mothers to the private over the public domain. This binary division of private from public marginalizes mothers, particularly those less privileged. The physical and social environments available to new mothers/parents then feel intimidating; the unease in public to managing babies, prams and bodily fluids become barriers to leaving the house (Boyer, 2012; Boyer and Spinney, 2016; Lugosi *et al.*, 2016). Transforming more public spaces into ‘third places’, places of connection, belonging and ease (Fullagar, 2019), could initiate systems of support in which the barriers of being in public are eliminated whilst maximizing the benefits of social connection in early motherhood. An exemplary model of such a third place is an indigenous-designed, mother-oriented hub, ‘Mamia’ in Aotearoa, in which mothers are welcomed and cared for in a space that was created to ‘feel like home’ (Lawrence, 2020).

Alongside physical environments, social environments are also crucial for fostering maternal health and reducing the likelihood of postpartum depression (Vaezi *et al.*, 2019). Organized social structures such as playgroups and social networks also promote maternal physical, mental and social health (Strange *et al.*, 2017; McLean *et al.*, 2020). Strange *et al.* (2014) show that for families with young children, the ability to form social networks is often dependent on community groups to provide social opportunities and that local community groups are helpful to parents with mobility problems. However, population-level maternal social support interventions have proven challenging to implement (Small *et al.*, 2014). Small *et al.* also note that often social support is embedded in a broader agenda (e.g. target ‘at risk’ mothers with a hidden curriculum of health education) that undermines a core purpose of building a social connection. Further, our suggestion of nesting a collective village into a maternal health promotion strategy also emphasizes physical environments alongside embedded support mechanisms that rely not only on mothers supporting mothers but also on the broader community to acknowledge and care for mothers/parents during this vulnerable time.

Inherently it is impossible in 2022 to think about a ‘village’ in motherhood without a notion of social media, as it has a pivotal role in early motherhood (Baker and Yang, 2018; Pretorius *et al.*, 2019). Indeed, participants highlighted the benefits *women supporting women online* could have for connecting those with similar interests. Archer and Kao (2018) found that with the decline of local support networks, social media can provide essential mother-to-mother support; however, caution on the overreliance on technology for social support is warranted (Ginja *et al.*, 2018). Online support can enhance, facilitate and strengthen bonds, particularly parenting practices or needs, and should be included in a comprehensive maternal health promotion approach.

Additional ideas that resonated with a broader theme of building a village included *intergenerational support and connections*. Participants desired to *include all members of society* and *connect the elderly with young families*, and references to existing organizations such as ‘*supergrans*’ were made. Indeed, intergenerational support mechanisms that go beyond kin have the potential to foster well-being across families and older community members (Szabó *et al.*, 2021). *Peer support programmes* were also suggested as mechanisms for early emotional support. Participants wanted peer support to be *local* and *funded* for equity of access. Peer support programmes such as volunteer home visiting programmes (Byrne *et al.*, 2016) or volunteer doula support (McLeish and Redshaw, 2019b) can promote maternal emotional well-being, self-esteem and self-efficacy and contribute to a multi-level approach promoting maternal health.

### Strengths and limitations

This research drew on a large diverse maternity stakeholder group from urban and rural geographies in Aotearoa, enabling breadth and diversity of perspectives. The explicit theoretical and values-based orientation offered a means to envision a more comprehensive, upstream and coordinated approach to maternal health promotion beyond an emphasis on biomedical and lifestyle factors. Limitations of this research include its broad scope and limited depth of engagement with the participants. The approach covered broad grounds and was not able to engage in too much depth with any of the ideas in the workshops, nor in the discussion of the findings in this article. However, we see this as an opportunity provide a big picture of what is possible (and more) if we can ground our work in a mother-centred approach.

## CONCLUSION

Current maternal health promotion in Aotearoa is fragmented and inefficient for meeting needs, likely much of which is evident in maternal health promotion across the globe. Maternal health statistics in Aotearoa (PMMRC, 2021) and globally (Crear-Perry *et al.*, 2021) show increasing disparities. The lack of a comprehensive maternal health promotion strategy built on health promotion values and models has the potential to bring together existing work and build a vision for new comprehensive approaches across individual, community and policy levels. Maternity stakeholders in this research proposed a broad scope of opportunities that could positively promote maternal health, including building connected systems close to home, developing mothering/parenting skills, addressing upstream determinants, mother-centred care and funding, and creating a collective mothering village. Such a strategy could acknowledge the unique challenges of transitioning to motherhood and consider intersectional inequities in health. Rather than focussing only on individual behaviours, many ideas involved broader environmental and structural determinants. It was also apparent that the proposed framework is not a utopian wish but incorporates tenets of many initiatives and approaches already adopted, as evidenced in the literature. A comprehensive maternal health strategy could pull these strands together to provide a vision and pathway. Models to promote maternal health more comprehensively are promising (Fahey and Shenassa, 2013; Vogels-Broeke *et al.*, 2020), yet many lack coordinated health promotion strategising.

It is also worth noting that maternity care was mostly absent from the data, indicating that this part of the system was more satisfactory to participants. It is crucial to note that whilst community-based midwifery care is more able to meet equity needs (Neely *et al.*, 2020), the current workforce is understaffed and underfunded. It can this be difficult for women/pregnant people to find a midwife if they wait too long (Priday, 2018). Continued efforts to fund adequate maternity care is a core building block of maternal health promotion.

Informed by core health promotion values (equity, social justice, holistic health, socio-ecological, salutogenic, honouring Māori/indigeneity), gender-transformative health promotion (Pederson *et al.*, 2014), intersectional feminism (Zufferey and Buchanan, 2020) and matricentric feminism (O’Reilly, 2016) a comprehensive maternal health promotion strategy has the potential to enable, mediate and advocate for maternal health. It remains critical to decolonize health promotion theory and practice and engage in tools beyond the Ottawa Charter (McPhail-Bell *et al.*, 2013, 2019) to avoid perpetuating health inequities. Models of Indigenous maternal health promotion originate in different epistemologies. However, insights gained from this work may still be useful for thinking about how Indigenous maternal health promotion intersects with Western health system potentialities. Considering health holistically, thinking socio-ecologically, drawing on strengths, pursuing equity and social justice, and fostering Indigenous leadership are a few starting points for orienting maternal health promotion. We invite readers to contribute to and further build on our proposed maternal health promotion framework and publish their work as mother-centred health promotion to collectively advance a research agenda advocating for motherhood as a social good.
